# Multi-Omics Insights into the Effects of Long-Term Faba Bean Feeding on Muscle Quality and Metabolic Reprogramming in Nile Tilapia (*Oreochromis niloticus*)

**DOI:** 10.3390/ijms262210819

**Published:** 2025-11-07

**Authors:** Rongni Li, Saisai Wang, Yansheng Sun, Xin Zhang

**Affiliations:** Fisheries Science Institute, Beijing Academy of Agriculture and Forestry Sciences, Beijing 100068, China; wangsaisai@baafs.net.cn (S.W.); sunyansheng@baafs.net.cn (Y.S.)

**Keywords:** tilapia, 60% faba bean-based diet, effects of long-term feeding, meat quality assessment, histological analysis, transcriptome-metabolome integration, *chac1* gene, muscle hyperplasia

## Abstract

While short-term faba bean feeding is known to improve fish texture, its long-term systemic effects and the underlying molecular mechanisms in Nile tilapia remain rarely explored. This knowledge gap is critical, given the potential for extended feeding to induce distinct metabolic reprogramming and trade-offs. Here, we present the first comprehensive study investigating the 180-day impact of a 60% FB-based diet (FBD) on Nile tilapia through an integrated analysis of phenotypic traits, muscle histology, metabolome, and transcriptome. Our results revealed a fundamental trade-off: FBD feeding induced hyperplasia-driven muscle remodeling, significantly enhancing textural properties (hardness, gumminess, chewiness) and increasing intramuscular fat and collagen content, but at the cost of suppressed growth and hepatosomatic index. Metabolomics identified 243 significantly altered metabolites, outlining a systemic metabolic shift characterized by activated lipid synthesis but inhibited amino acid and energy metabolism. This multi-omics integration nominated the *chac1* gene as a novel key regulator for FB-induced muscle hyperplasia, a finding not previously reported in this context. We propose a mechanistic model wherein long-term FBD feeding coordinates lipid deposition, collagen accumulation, and *chac1*-mediated hyperplastic growth to remodel muscle texture. Our work provides new insights into the long-term metabolic trade-offs and molecular drivers of FB-induced flesh quality improvement, offering a theoretical foundation for developing optimized aquafeeds.

## 1. Introduction

Nile tilapia (*Oreochromis niloticus*) is a cornerstone of global aquaculture, ranking as the second most economically important farmed fish species worldwide [1]. Its significant contribution to food security is matched by its high demand in the international market [2]. However, the intensification of aquaculture practices has led to emerging challenges in the meat quality of tilapia, notably a loose texture and poor processing suitability [3]. These issues, manifesting as fragmentation and suboptimal taste in products like filets and fish balls, diminish the added value and market competitiveness of tilapia.

In response, dietary strategies using faba beans have emerged as a promising solution. A growing body of research has demonstrated that faba bean-based diets can significantly enhance the muscle quality of Nile tilapia by modifying muscle fiber structure, increasing collagen content, and regulating lipid metabolism [3,4]. Consequently, faba bean supplementation has become a crucial method for improving tilapia meat quality and remains a focal point in aquaculture research.

However, existing studies have primarily centered on the short-term (not exceeding 120 days) effects of faba beans on the growth performance or specific meat quality parameters of Nile tilapia [3,5,6,7], and have rarely employed multi-omics analyses. For instance, studies like Peng et al. [8], conducted 100-day trials but lacked multi-omics analyses. Conversely, other research, such as Xiaogang He et al. [5], applied transcriptomics and metabolomics after a 90-day feeding period but did not systematically correlate these molecular findings with comprehensive phenotypic and meat quality assessments. These studies lack systematic and in-depth multi-omics comprehensive analyses. Therefore, a thorough investigation into the long-term effects of faba bean feeding on the growth, meat quality, and molecular regulatory mechanisms of Nile tilapia is essential.

To address these gaps, we conducted a long-term (180-day) feeding trial. This study integrates high-throughput transcriptomic and metabolomic technologies with detailed phenotypic and histological observations. Our objectives are threefold: (1) to comprehensively evaluate the effects of long-term faba bean feeding on the muscle quality of Nile tilapia, with direct relevance to commercial value and practical application; (2) to uncover the mechanistic basis of faba bean-induced muscle quality improvement, particularly the formation of the desirable “crispness” trait; and (3) to identify key molecular players linking metabolic shifts to muscle fiber hyperplasia. Elucidating these mechanisms will not only advance the theoretical understanding of muscle biology in teleost fish but also pave the way for developing innovative, sustainable feeding strategies to enhance the quality and marketability of Nile tilapia products.

## 2. Results

### 2.1. Phenotypic and Compositional Analyses

#### 2.1.1. Growth, Morphometry, and Organ Indices

Following the six-month feeding trial, significant differences in growth and morphometric parameters were observed between the control (C) and experimental (E) groups (Table 1). Fish fed the faba bean-based diet (E) displayed significantly lower final body weight (BW), total length (TL), condition factor (CF), and hepatic somatic index (HSI) compared to the C group (all *p* < 0.05). In contrast, the intestinal fat rate (IFR) was markedly elevated in group E (*p* = 0.022). No significant differences were detected in standard length (SL), filet yield (FY), visceral somatic index (VSI), or intestinal somatic index (ISI) between the two groups (*p* > 0.05).

#### 2.1.2. Muscle Histology and Physicochemical Properties

**Figure 1:**
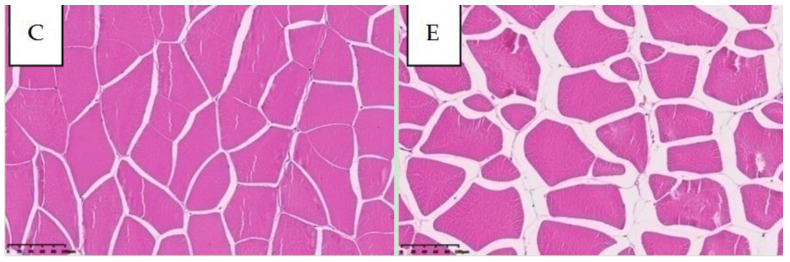
Comparative cross-sectional micrographs of dorsal muscle tissue in Nile tilapia (*Oreochromis niloticus*) from groups C and E. Scale bar: 100 μm.

Significant alterations in muscle microstructure and physicochemical properties were identified in group E compared to group C (Table 2, Figure 1). The muscle fiber density and steaming loss were significantly higher in group E (*p* < 0.01), whereas the muscle pH was significantly lower (*p* < 0.01). Cross-sectional histological examination further revealed that muscle fibers in group E possessed substantially larger intermyofibrillar spaces than those in group C (Figure 1).

#### 2.1.3. Muscle Textural Properties

Texture profile analysis demonstrated substantial differences between the dietary groups (Table 3). Muscle samples from group E exhibited significantly greater hardness, gumminess, and chewiness (all *p* < 0.001) compared to group C. Conversely, adhesiveness (*p* = 0.002) and springiness (*p* = 0.009) were significantly lower in group E.

#### 2.1.4. Muscle Nutritional Composition

Comparative analysis of muscle composition revealed significant differences between groups C and E (Table 4 and Table 5). Group E exhibited significantly lower ash (*p* = 0.001) and phosphorus (*p* = 0.017) content, alongside markedly higher crude fat (*p* = 0.012) and collagen protein (*p* = 0.032) levels. No significant inter-group differences were observed in moisture, crude protein, energy, or calcium content (Table 4).

Amino acid profiling indicated a significant reduction in the total content of the 18 analyzed amino acids in group E (*p* = 0.018). Among these, 12 amino acids—including five key umami-taste amino acids (glutamic acid, glycine, aspartic acid, alanine, and arginine)—were significantly decreased in group E (*p* < 0.05). In contrast, the levels of threonine, serine, proline, cystine, valine, and tryptophan remained unchanged (*p* > 0.05) (Table 5).

### 2.2. Muscle Metabolomic Profiling

#### 2.2.1. OPLS-DA Modeling and Validation

**Figure 2:**
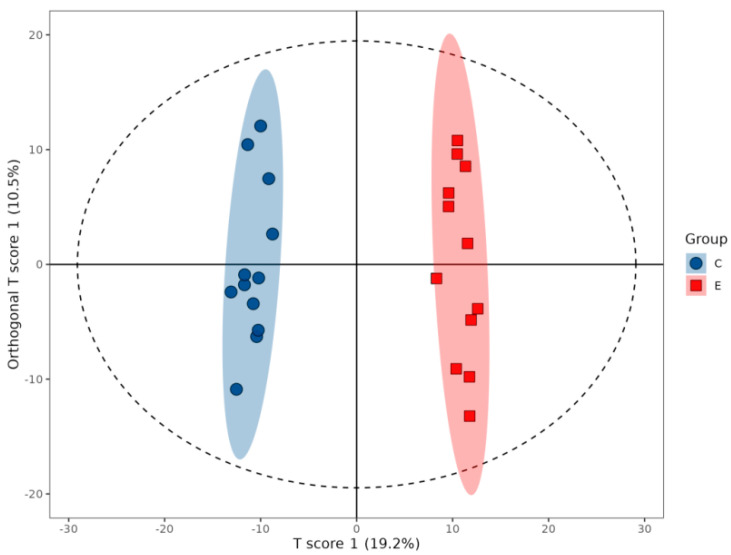
OPLS-DA score plot for muscle metabolomes of Nile tilapia fed control (C, blue) versus faba bean-supplemented (E, red) diets.

Orthogonal projections to latent structures-discriminant analysis (OPLS-DA) was applied to characterize the metabolic perturbations induced by the faba bean diet. The model effectively discriminated between groups C and E, with cumulative parameters (R^2^Y = 0.988, Q^2^ = 0.925) indicating high explanatory power (98.8% variance) and predictive reliability (Q^2^ > 0.5). Distinct clustering of samples along the predictive component confirmed clear separation between groups and homogeneity within groups (Figure 2).

**Figure 3:**
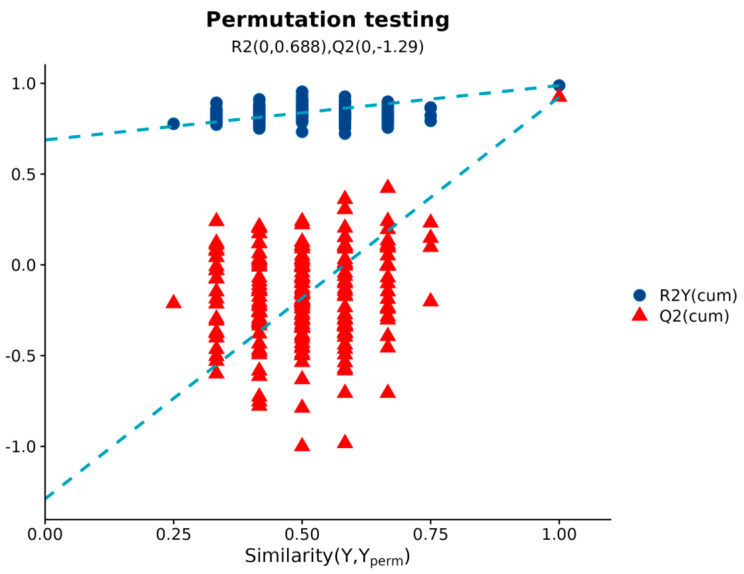
Permutation test results (*n* = 200 iterations) for OPLS-DA model validation. The dashed line represents the regression slope of permuted Q^2^ values.

A 200-permutation test validated the model’s robustness, yielding intercepts of R^2^ = 0.688 and Q^2^ = −1.29 (Figure 3). The negative Q^2^ intercept (<0) and the uniformly lower permuted Q^2^ values relative to the original model confirmed a minimal risk of overfitting, consistent with established metabolomic validation criteria [9].

The raw metabolomics data were deposited in the OMIX database at the China National Center for Bioinformation/Beijing Institute of Genomics, Chinese Academy of Sciences (accession no. OMIX008551).

#### 2.2.2. Identification of Differentially Abundant Metabolites

**Figure 4:**
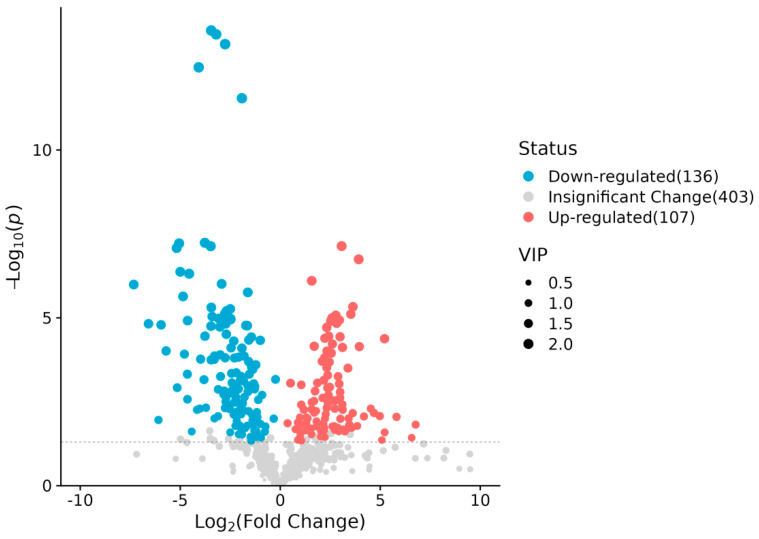
Volcano plot of differentially abundant metabolites in E vs. C groups. Red/blue dots denote upregulated/downregulated metabolites (*VIP* > 1, *p* < 0.05). Dashed vertical line: *p* = 0.05 threshold.

Differential metabolites were identified using thresholds of variable importance in projection (VIP) > 1 and *p* < 0.05 (Student’s *t*-test). A total of 243 significantly altered metabolites (SAMs) were identified between groups E and C, comprising 107 upregulated and 136 downregulated compounds (Figure 4).

The top 10 SAMs, ranked by VIP score, were as follows: Arachidonic Acid (ARA) was upregulated in group E; the other nine metabolites were downregulated: 3-formylindole, 2-piperidone, 4-methyl-5-thiazoleethanol, 6-hydroxy-7-methoxycytophycin E, simvastatin, panaxydiol, valine betaine, O-propanoylcarnitine, and 17α-hydroxyprogesterone.

#### 2.2.3. Integrated Metabolic Pathway Analysis

**Figure 5:**
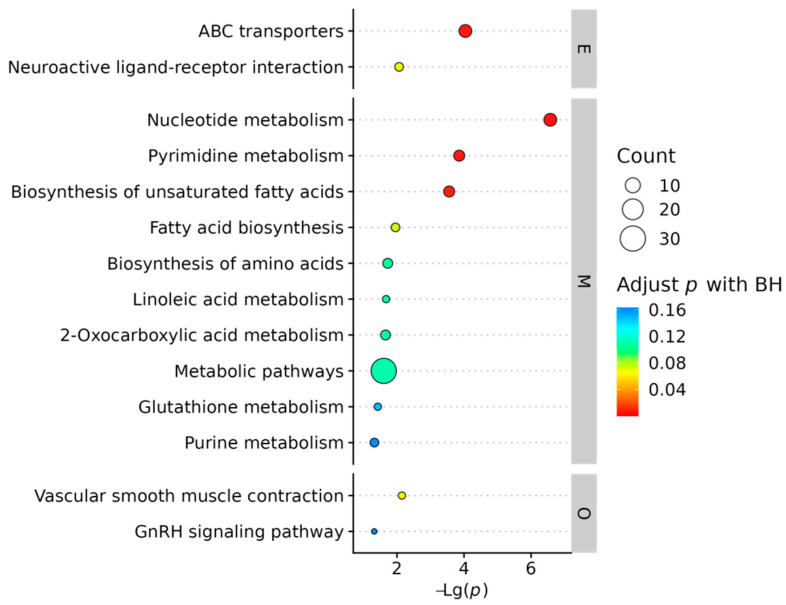
KEGG pathway enrichment bubble map for E vs. C groups. Note: KEGG Level 1 classifications Metabolism (M), Environmental Information Processing (E), and Organismal Systems (O). Bubble size reflects the number of annotated metabolites (count), while color intensity corresponds to the significance level (−*log*10 (*p*-value)).

KEGG pathway enrichment analysis based on the SAMs was performed to investigate systemic metabolic alterations. Significantly enriched pathways were identified using a threshold of corrected *p*-value < 0.05 (Figure 5). The top 30 enriched pathways were categorized under KEGG Level 1 classifications, with nucleotide metabolism, ABC transporters, pyrimidine metabolism, and biosynthesis of unsaturated fatty acids being the most prominent.

**Figure 6:**
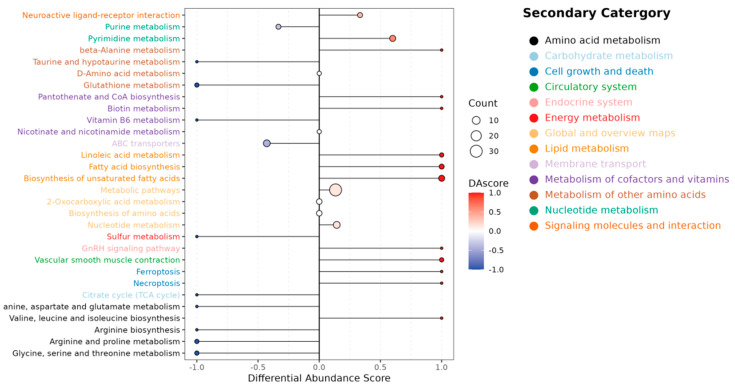
DA score map of differentially abundant metabolites. (E vs. C). Note: *X*-axis: DA score. *Y*-axis: pathways. DA scores range from −1 ([blue], all metabolites downregulated) to +1 ([red], all upregulated), with color gradients (blue to red) and circle sizes indicating score magnitude and metabolite count, respectively.

To evaluate the global directionality of metabolic changes, a differential abundance (DA) score was calculated to quantify the collective perturbation of metabolites within each pathway (Figure 6). Key observations included: Amino Acid Metabolism: Six out of nine pathways (e.g., arginine biosynthesis, glutathione metabolism) were downregulated; Lipid Metabolism: All three assessed pathways (linoleic acid metabolism, fatty acid biosynthesis, biosynthesis of unsaturated fatty acids) exhibited upregulated trends.; Energy Metabolism: Pathways related to sulfur metabolism and the TCA cycle were suppressed; Circulatory System: Vascular smooth muscle contraction was upregulated, suggesting a potential systemic physiological adaptation.

These findings indicate coordinated metabolic shifts, characterized by activated lipid anabolism alongside suppressed amino acid and energy-related pathways.

**Figure 7:**
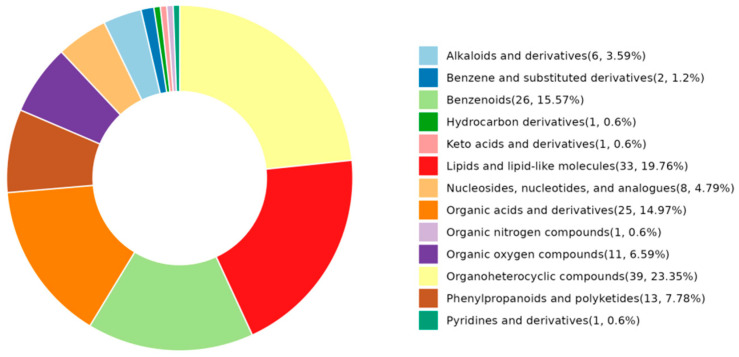
HMDB class circular diagram of SAMs.

HMDB Super Class analysis categorized the SAMs into five major classes (Figure 7): Organoheterocyclic compounds (23.25%), Lipids and lipid-like molecules (19.76%), Benzenoids (15.57%), Organic acids and derivatives (14.97%), and Phenylpropanoids and polyketides (7.78%). Collectively, these categories accounted for 81.33% of all annotated metabolites. This profile suggests significant modifications in biochemical pathways related to lipid metabolism and secondary metabolite synthesis in response to the dietary intervention.

### 2.3. Muscle Transcriptome Analysis

#### 2.3.1. Sequencing and Data Quality

Transcriptomic profiling was conducted to assess mRNA expression differences. A total of 50.54 GB of raw sequencing data were generated, with 47.72 GB of high-quality clean data retained after filtering (clean read ratio > 92%, Q30 > 94.2%). All raw data were deposited in the Genome Sequence Archive (GSA: CRA021826). Read alignment to the *Oreochromis niloticus* reference genome (NCBI Assembly ID: 391053) yielded a mapping rate > 93.5%, with 97.9–98.2% of reads mapped to exonic regions (Table 6), confirming data reliability for subsequent analysis.

#### 2.3.2. Sample Correlation and Differential Expression

**Figure 8:**
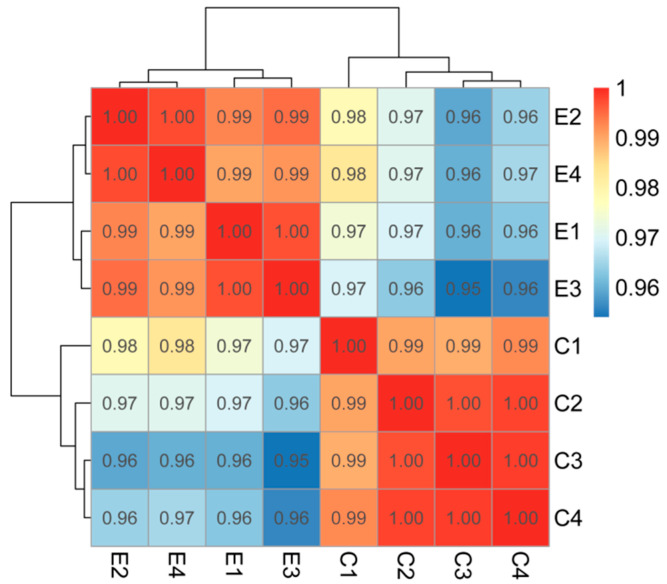
Heat map of the correlation coefficient (method = pearson) between samples. Note: The left and top sides show the clustering of samples, while the right and bottom sides show the sample names. Different colored squares represent the correlation strength between two samples.

Pearson correlation analysis demonstrated high intra-group reproducibility (r > 0.99) and clear separation between groups C and E (Figure 8), validating experimental reliability.

**Figure 9:**
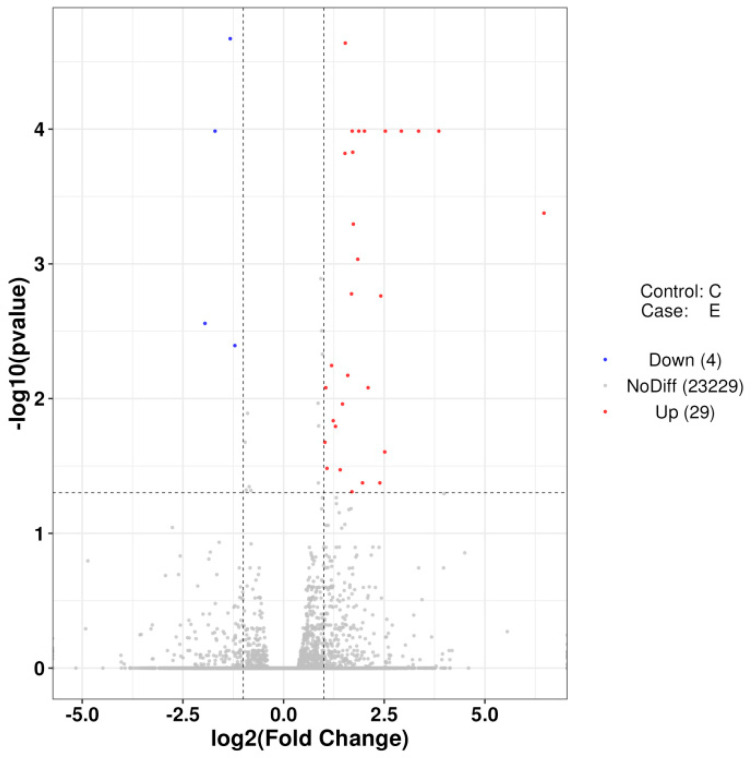
Volcano plot of DEGs identified with groups C and E.

**Figure 10:**
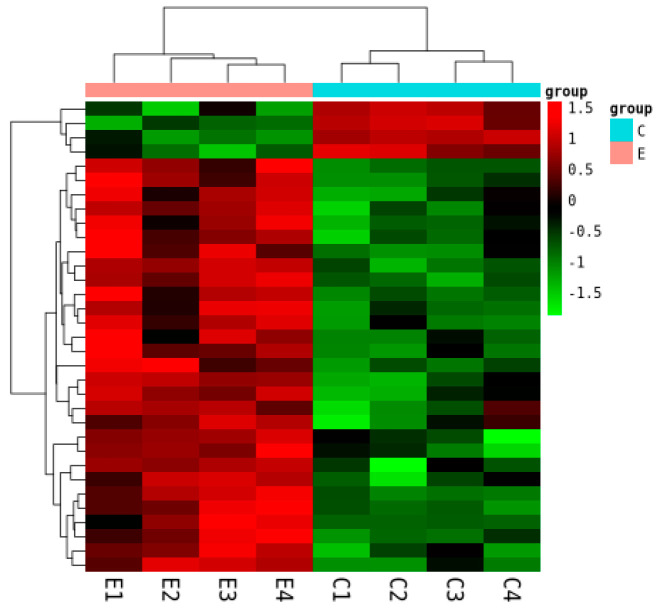
Hierarchical clustering heatmap of DEGs between groups C and E.

Differential expression analysis identified 33 significantly differentially expressed genes (DEGs) (|*log*2 (*fold change*)| > 1, *FDR* < 0.05). Of these, 29 were upregulated and 4 were downregulated in group E (Figure 9). Hierarchical clustering of these DEGs showed consistent intra-group expression patterns and clear inter-group separation (Figure 10).

#### 2.3.3. Functional Enrichment of DEGs

**Figure 11:**
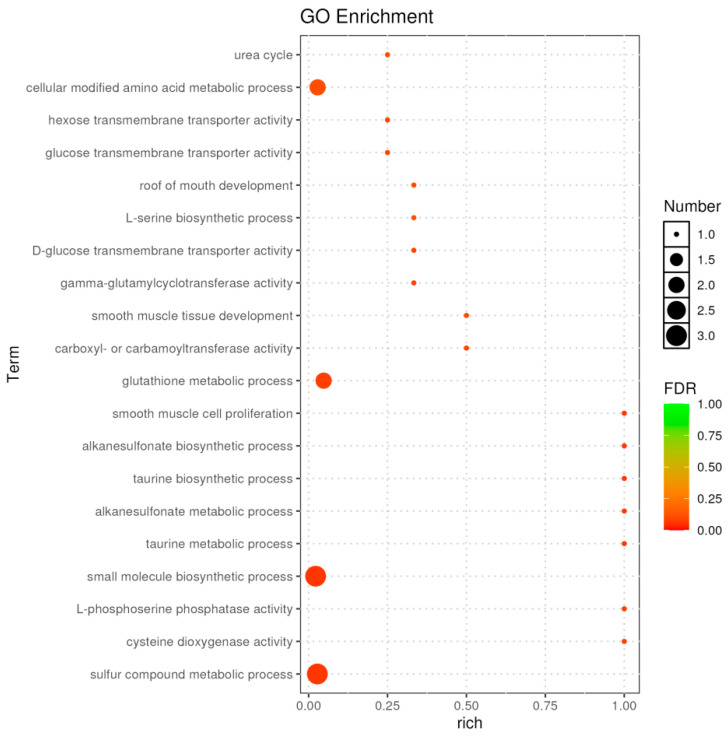
GO classification analysis of DEGs between C vs. E.

Gene Ontology (GO) enrichment analysis of the DEGs revealed significant enrichment in terms related to sulfur compound metabolism, glutathione metabolism, and small molecule biosynthesis (Figure 11). Key genes involved included *cdo1*, *chac1*, *LOC100695721*, *LOC100706939*, and *psph*.

**Figure 12:**
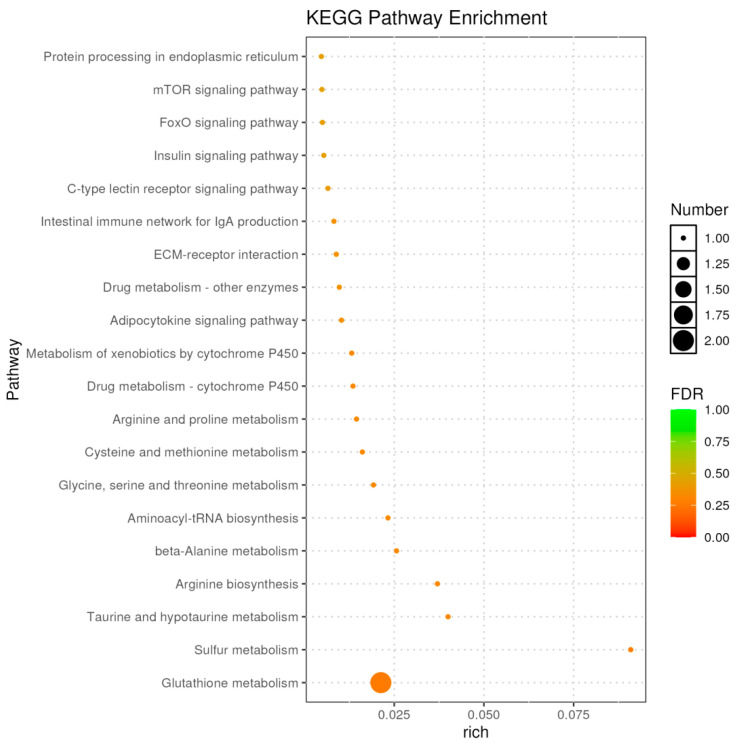
KEGG classification analysis of DEGs between C vs. E.

KEGG pathway analysis further corroborated these findings, identifying significant enrichment (*FDR* < 0.05) in glutathione metabolism, taurine and hypotaurine metabolism, and sulfur metabolism (Figure 12). Key regulators in these pathways included *chac1*, *LOC100695721*, *cdo1*, and *LOC100698874*. These results suggest that the faba bean diet induces significant transcriptional changes in tilapia muscle, primarily affecting sulfur and glutathione-related metabolic pathways.

### 2.4. RNA-Seq Data Validation

**Figure 13:**
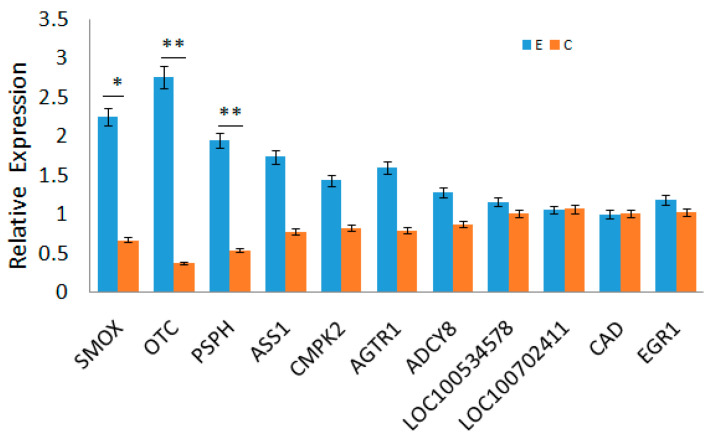
Verification of mRNA Expression Levels by qPCR. Note: The blue bars represent the group E, while the brown bars represent the group C. Statistical significance is indicated as follows: * *p* < 0.05, ** *p* < 0.01.

To validate the RNA-Seq results, the expression of 16 randomly selected genes was analyzed by qRT-PCR. The expression patterns determined by qPCR were consistent with the RNA-Seq data (Figure 13), confirming the reliability of the transcriptomic findings.

### 2.5. Integrated Transcriptomic and Metabolomic Analysis

**Figure 14:**
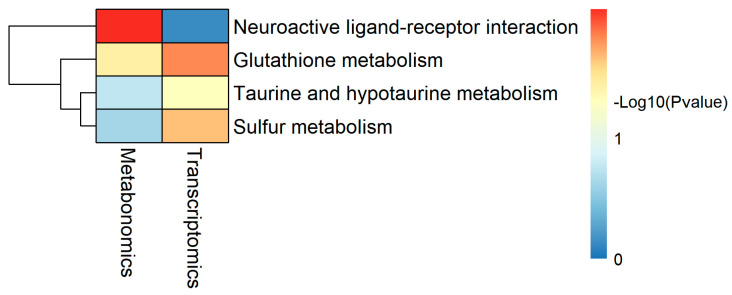
*p*-value_heatmap of Shared KEGG pathways between Ts and Ms.

A joint KEGG pathway enrichment analysis was performed to integrate the transcriptomic and metabolomic datasets. Shared pathways were identified and visualized (Figure 14, Table 7). Notably, four DEGs (*chac1*, *LOC100695721*, *cdo1*, *LOC100698874*) and three DAMs (ARA, Ornithine, Taurine) were consistently associated with the top co-enriched pathways. The upregulation of *chac1* and *cdo1*, coupled with the downregulation of *LOC100695721* and the metabolites ARA, Ornithine, and Taurine, suggests a coordinated molecular response to long-term faba bean supplementation. These findings implicate glutathione homeostasis, sulfur cycling, and taurine metabolism as potential regulatory hubs linking the dietary intervention to the observed changes in muscle quality.

## 3. Discussion

### 3.1. Long-Term Faba Bean Feeding Improves Muscle Composition and Texture

Our 180-day feeding trial demonstrates that a faba bean-based diet (FBD) significantly enhances tilapia muscle quality, primarily through improvements in chemical composition and physical texture—two distinct but interrelated aspects.

Regarding chemical composition, intramuscular fat content in the E reached 2.02%, significantly higher than the C (1.30%) and aligning with the 2% benchmark for optimal palatability in meats like pork [10]. This value also surpasses levels reported in shorter-term faba bean studies [11], underscoring the long-term effect. Metabolomic data corroborated this finding, showing an upregulation of lipid synthesis pathways. Concurrently, collagen content was significantly elevated. As a key structural protein, collagen contributes to muscle integrity and mechanical strength [12], and its increase is directly associated with greater hardness in fish muscle [13].

These compositional changes underpin the observed textural improvements. Texture profile analysis revealed a significantly stronger muscle matrix in the E, characterized by increased hardness, gumminess, and chewiness, alongside reduced adhesiveness and springiness. This profile suggests a material that requires greater initial chewing force but exhibits less elastic recovery, contributing to a “crisp yet not tough” sensation. The increase in hardness is particularly valuable for aquatic species, which typically have softer muscle than terrestrial livestock, limiting their processing potential [14]. Finally, increased muscle fiber density, which correlates with hardness and chewiness [15], likely acted synergistically with higher collagen levels to reinforce the muscle texture.

In summary, long-term FBD feeding enhanced both the biochemical constituents and the physical structure of tilapia muscle, collectively improving its mechanical strength and processing suitability.

### 3.2. Altered Muscle Metabolome and Potential Flavor Implications

Metabolomic analysis revealed that long-term FBD feeding profoundly reshaped the muscle metabolome. Among the top 10 significantly altered metabolites were compounds with implications for nutrition and flavor.

The elevation of arachidonic acid (ARA), an essential fatty acid [16], suggests an improved nutritional profile. Conversely, the reduction of 17α-hydroxyprogesterone, a steroid hormone precursor [17], may lower the risk of hormonal residue accumulation, aligning with food safety standards. The downregulation of 2-piperidone, a lysine-derived metabolite [18], suggests suppressed protein degradation [19], potentially aiding in the maintenance of muscle firmness.

Notably, the decrease in flavor-active metabolites like 4-methyl-5-thiazoleethanol (associated with sulfur-like off-odors) and 3-formylindole (linked to bitterness) [20] suggests a mitigation of undesirable flavors. The reduction of 6-hydroxy-7-methoxyscytophycin E, a structural analog of cyanobacterial toxins, may further reduce potential algal-derived off-flavors. Collectively, these metabolic shifts point toward a cleaner and more pleasant sensory profile.

### 3.3. Adverse Effects on Growth Performance and Underlying Mechanisms

Consistent with some previous studies [7,11], FBD feeding significantly inhibited growth, suppressed liver development (evidenced by a lower hepatosomatic index), and increased mesenteric fat deposition. This growth inhibition may be attributed to reduced protein, energy, and fat digestibility when faba bean content exceeds 50% [21].

We hypothesize that taurine metabolism is central to the observed liver suppression. Taurine is primarily synthesized in the liver [22], and its deficiency is a known cause of growth inhibition. Our data show lower muscle taurine content in the E, and integrated pathway analysis highlighted taurine and hypotaurine metabolism as significantly enriched. The decreased hepatosomatic index aligns with findings that taurine can reduce liver weight under metabolic stress [23]. This effect could be due to either the low taurine content in faba beans or the presence of compounds that interfere with its synthesis.

Furthermore, FBD feeding increased muscle water loss rate and lowered muscle pH, adversely affecting water-holding capacity—a finding consistent with the general principle that lower pH reduces water retention in muscle [24]. The downregulation of valine betaine, a metabolite involved in osmoregulation [25], may partly explain this phenomenon.

### 3.4. Trade-Offs in Muscle Metabolism: Amino Acids, Lipids, and Energy

A notable trade-off was observed: while intramuscular fat increased, the total of free amino acids (FAAs) was reduced. This FAAs reduction, consistent with findings in other fish species [26,27], involved key umami-inducing amino acids (aspartic and glutamic acid) and most bitter-tasting amino acids, potentially altering the flavor profile by reducing both umami intensity and bitterness.

The discordance between reduced FAAs and stable crude protein content suggests a shift in nitrogen metabolism, possibly driven by the dietary composition [28]. The reduction of specific metabolites known to inhibit lipid accumulation—taurine [29,30], arginine [31], and simvastatin [32]—provides a plausible explanation for the concurrent increase in intramuscular fat deposition.

Metabolomic profiling also indicated a broad suppression of energy metabolism, including the TCA cycle and sulfur metabolism. The downregulation of citrate and multiple energy-yielding FAAs supports this finding, revealing a state of reduced energy turnover that aligns with reported adaptations to nutritional stress in teleosts [6].

### 3.5. A Hypothesized Model for FBD-Induced Muscle Remodeling

We propose a mechanistic model where long-term FBD intake induces muscle remodeling primarily through hyperplasia, leading to the improved texture (Figure 1). Fish muscle exhibits high plasticity, retaining the capacity for lifelong hyperplasia [33,34,35].

Our histological findings—increased muscle fiber density, larger fiber gaps, and irregular myofibril arrangement—collectively indicate hyperplasia-driven structural remodeling, consistent with reports in grass carp [36]. This process is metabolically demanding, requiring high amino acid utilization [37]. The observed reduction in total FAAs and the enrichment of amino acid catabolism pathways in our multi-omics data likely reflect this increased anabolic demand for mosaic hyperplasia.

The remodeling process appears to be coordinated by specific metabolites and a key regulatory gene. The significant upregulation of ARA, a lipid known to promote in vitro muscle cell growth [38], and the accumulation of collagen, which regulates cell proliferation during tissue morphogenesis [39], are both implicated. Integrated transcriptomic and metabolomic analysis nominated the *chac1* gene as the most significant regulator. *Chac1* expression is upregulated during myoblast differentiation in other species [40,41], and its enhanced expression in the E, coupled with the histological evidence of hyperplasia, strongly suggests it promotes myoblast proliferation and differentiation in tilapia, facilitating muscle remodeling. The rationale for selecting the *chac1* gene is detailed below.

### 3.6. The chac1 Gene as a Key Regulator in FBD-Induced Muscle Remodeling

Integrated transcriptomic and metabolomic analyses identified four DEGs: *chac1*, *LOC100695721* (GSTA), *LOC100698874* (ETHE1), and *cdo1*. Among these, *cdo1* serves as a rate-limiting enzyme in taurine biosynthesis [22], linking it to the observed growth inhibition. The other two genes (*LOC100695721* and *LOC100698874*) are involved in glutathione metabolism and cellular homeostasis [42,43]; however, direct evidence of their role in myogenesis is currently lacking.

In contrast, the function of the *chac1* gene is closely related to myogenesis. As a member of the γ-glutamyl cyclotransferase family, *chac1* is upregulated under developmental and stress conditions [44]. Multiple studies support its direct role in muscle development: *chac1* expression is significantly upregulated during myoblast differentiation, and its functional manipulation directly promotes myoblast proliferation and differentiation [40]. A role for *chac1* has also been conserved in the myogenic process of Atlantic salmon muscle [41].

In this study, transcriptomic analysis revealed a significant enhancement of *chac1* expression in the E. This upregulation, coupled with our histological evidence of increased muscle fiber density—a hallmark of hyperplasia—strongly suggests that *chac1* promotes the proliferation and differentiation of myoblasts in tilapia. These results support the hypothesis that long-term FBD feeding induces the upregulation of *chac1* expression in muscle tissue, which in turn acts as a significant molecular regulator driving myoblast hyperplasia, thereby facilitating the observed muscle remodeling and texture improvement. Consequently, *chac1* is nominated as a key candidate gene mediating the muscle quality improvements in tilapia fed a faba bean-based die

In conclusion, long-term FBD feeding induces a complex metabolic reprogramming in Nile tilapia. It triggers a hyperplastic remodeling of muscle tissue, coordinated by metabolites like ARA and collagen and likely driven by the *chac1* gene, resulting in improved textural properties. These benefits are accompanied by trade-offs, including growth inhibition, altered flavor compound profiles, and reduced energy metabolism, which warrant consideration in practical applications.

## 4. Materials and Methods

### 4.1. Experimental Diets, Fish Culture, and Tissue Collection

Juvenile genetically improved farmed tilapia (GIFT, *Oreochromis niloticus*) were obtained from the Xiaotangshan Breeding Base of the Fisheries Science Institute, Beijing Academy of Agriculture and Forestry Sciences. The entire feeding trial was conducted at this facility.

#### 4.1.1. Experimental Design and Fish Rearing

A total of 400 GIFT fry (initial average body weight: 309.98 ± 11.88 g) were randomly allocated into two dietary groups: a control group (C) and an experimental group (E). Each group was assigned to three replicates. The control group was fed a commercial tilapia extruded feed (P522, Tianjin Tianshinong Agricultural Technology Co., Ltd., Tianjin, China.), while the experimental group received a specially formulated diet containing 60% faba bean of the total feed mass (FBD, “Feicui No. 1-1777”, Zhongshan Taishan Feed Co., Ltd., Zhongshan, China). The proximate composition of both diets is presented in Table 8.

#### 4.1.2. Culture System and Environmental Management

The experiment was conducted in a transparent-roofed indoor shelter housing concrete tanks (5.2 m × 2.8 m × 0.87 m) with a flow-through water system. Each tank was equipped with a bottom nanobubble disc aerator to maintain dissolved oxygen levels. To create three replicates per dietary group, each tank was divided into three equal compartments using fixed separating nets, stocking approximately 67 fish per compartment (200 fish per tank). The water source was groundwater. Approximately one-third of the water in each tank was replaced daily via draining to manage waste. Key water quality parameters, monitored regularly, were maintained within the following ranges: pH 7.0–8.4, dissolved oxygen > 5.0 mg/L, nitrite < 0.02 mg/L, and ammonia nitrogen < 0.15 mg/L. The trial was conducted under natural light and ambient temperature (25–32 °C).

#### 4.1.3. Feeding Regime and Duration

The fish were hand-fed to apparent satiety twice daily (at 08:00 and 14:00), with the feeding amount adjusted to ensure all feed was consumed within five minutes. The entire feeding trial lasted for 180 days, from 12 April to 10 October 2021.

Prior to tissue sampling, the fish were fasted for 24 h and then anesthetized with tricaine methanesulfonate (MS222, 100 mg/L). Subsequently, 20 fish from each group (C and E) were randomly selected after six months of breeding. Among them, six individuals per group were subjected to morphometric measurements. Muscle samples were collected from the area between the dorsal fin and lateral scales of each fish (The dorsal muscle constitutes the largest and most economically valuable portion of the tilapia meat. Assessing the dorsal muscle allows our findings to be directly translated into practical outcomes.). Part of the muscle samples were flash-frozen in liquid nitrogen and then stored at −80 °C for subsequent transcriptome, metabolome, and RT-qPCR analyses. The remaining muscle samples were used for texture analysis and nutritional composition assessment.

### 4.2. Assessment of Growth Performance, Nutritional Composition, and Histomorphological Parameters

#### 4.2.1. Quantification of Morphometric Indices

Six fish per group (C and E) were subjected to morphometric analyses. Body weight (*BW*), standard length (*SL*), and total length were measured prior to dissection. Visceral mass, liver weight, intestinal weight, and abdominal adipose tissue were individually weighed. Dorsal-ventral muscle tissues were excised, deboned, and skinned to determine muscle mass. The following indices were calculated:

*Adipose somatic index* (%) = 100 × (*abdominal adipose weight*/*BW*)

*Filet yield* (%) = 100 × (*muscle mass*/*BW*)

*Viscerosomatic index *(%) = 100 × (*visceral mass*/*BW*)

*Hepatosomatic index* (%) = 100 × (*liver weight*/*BW*)

*Intestinal somatic index* (%) = 100 × (*intestinal weight*/*BW*)

*Condition factor *(%) = 100 × (*BW*/*SL*^3^)

#### 4.2.2. Proximate Composition Analysis of Muscle Tissue

Muscle samples (*n* = six replicates/group) were analyzed for nutritional constituents using standardized methods:

Moisture content: GB5009.3-2016 [45] (Method I, gravimetric oven drying)

Ash content: GB5009.4-2016 [46] (Method I, muffle furnace combustion)

Crude protein: GB5009.5-2016 [47] (Method I, Kjeldahl nitrogen analysis)

Crude lipid: GB5009.6-2016 [48] (Method II, Soxhlet extraction)

Gross energy: GB/Z 21922-2008 [49] (bomb calorimetry)

Calcium (Ca) and phosphorus (P): GB5009.268-2016 [50] (Method II, ICP-OES)

Amino acid profile: Aspartic acid, threonine, serine, glutamic acid, proline, glycine, alanine, cystine, valine, methionine, isoleucine, leucine, tyrosine, phenylalanine, lysine, histidine, arginine: GB5009.124-2016 [51] (HPLC).

Tryptophan: GB/T 15400-2018 [52] (spectrophotometric assay).

Total amino acids: GB5009.124-2016 [51] & GB/T 15400-2018 [52] (hydrolysis-HPLC)

Collagen content: Quantified via hydroxyproline assay kit (Nanjing Jiancheng Bioengineering Institute, Nanjing, China) following the manufacturer’s instructions. Collagen content = Hydroxyproline concentration/0.11.

#### 4.2.3. Texture Profile Analysis and Physicochemical Characterization

Muscle samples stored at −20 °C (*n* = 12 replicates/group) were thawed at 4 °C for texture analysis. Texture parameters: Hardness, adhesiveness, springiness, gumminess, and chewiness were determined using an FTC TMS-Pro texture analyzer (Food Technology Corporation, Sterling, VA, USA) equipped with a SMS P/25 cylindrical probe (diameter = 25 mm). Samples (1 cm^3^ dorsal muscle cubes) were compressed twice to 50% strain at 30 mm/min in TPA mode. Data were processed via Texture Pro v1.18-208 software.

Cooking loss: Fresh muscle (≈3 g, W_1_) was steamed for 5 min, cooled, surface-dried, and reweighed (W_2_). Cooking loss (%) = 100 × (W_1_ − W_2_)/W_1_.

pH: Measured electrometrically per GB5009.237-2016 (homogenate method).

#### 4.2.4. Histological Analysis and Muscle Fiber Density Determination in Tilapia Muscle Tissue

Muscle samples from the groups C and E were collected for histological analysis, with six replicates per group.

Muscle fiber density assessment: Hematoxylin-eosin (H&E) staining was performed on 3 mm thick muscle tissue sections following standard histological protocols. Muscle fiber density quantification was conducted using Image-Pro Plus 6.0 analysis software (Media Cybernetics, Rockville, MD, USA). Three randomly selected microscopic fields (standardized in millimeter units) were analyzed per histological section. The number of muscle fibers within each field was enumerated, accompanied by precise measurements of both field area and corresponding tissue area. Muscle fiber density was subsequently calculated using the formula: Density (fibers/mm^2^) = Total fiber count/Measured tissue area.

#### 4.2.5. Statistical Analysis of Growth, Nutritional, and Histological Parameters

Data are expressed as mean ± standard error (SE). Differences between the groups C and E were analyzed using an unpaired two-tailed Student’s *t*-test for normally distributed data. All statistical analyses were performed with Microsoft Excel 2007 (Microsoft Corp., Redmond, WA, USA). Statistical significance was defined at *p* < 0.05.

### 4.3. Non-Targeted Metabolomic Analysis

#### 4.3.1. Samle Preparation

Muscle tissues from liquid nitrogen-preserved groups C and E fish (*n* = 12 per group) underwent metabolomic profiling. Approximately 100 mg of each muscle sample was homogenized in 1000 μL of extraction solvent (water: methanol: acetonitrile = 1:2:2, *v*/*v*/*v*), and then placed for 1 h ultrasonic shaking in ice baths. Subsequently, the mixture was placed at −20 °C for 1 h and centrifuged at 14,000× *g* for 20 min at 4 °C. The supernatants were recovered and concentrated to dryness in vacuum.

#### 4.3.2. UHPLC-MS/MS Analysis

Metabolomics profiling was analyzed using a UPLC-ESI-Q-Orbitrap-MS system (UHPLC, Shimadzu Nexera X2 LC-30AD, Shimadzu, Japan) and analyzed using a Q-Exactive MS system (Thermo Scientific, San Jose, CA, USA) equipped with an ACQUITY UPLC BEH Amide column (100 mm × 2.1 mm, 1.7 μm; Waters, Milford, MA, USA). Chromatographic separation was achieved under the following conditions: column temperature 25 °C, flow rate 0.3 mL/min, and injection volume 3 μL. The mobile phase consisted of (A) 25 mM ammonium acetate aqueous solution and (B) acetonitrile, with gradient elution programmed as follows: 0–1 min (95% B), 1–7 min (95–65% B), 7–9 min (65–35% B), 9–10.5 min (35% B), 10.5–11 min (35–95% B), and 11–15 min (95% B).

Mass spectrometric detection was performed in both positive and negative ionization modes with optimized parameters: capillary temperature 320 °C, S-lens RF level 50, sheath gas flow 30 arb, auxiliary gas flow 5 arb, probe heater temperature 350 °C, and spray voltages of +3.8 kV (positive mode) or −3.2 kV (negative mode).

#### 4.3.3. Data Preprocessing and Filtering

The raw MS data were processed using MS-DIAL for peak alignment, retention time correction and peak area extraction. The metabolites were identified by accuracy mass (mass tolerance < 10 ppm) and MS/MS data (mass tolerance < 0.02 Da) which were matched with HMDB, massbank and other public databases. In the extracted-ion features, only the variables having more than 50% of the nonzero measurement values in at least one group were kept.

#### 4.3.4. Multivariate Statistical Analysis

R(version:4.0.3) and R packages were used for all multivariate data analyses and modeling. Data were mean-centered using Pareto scaling. Models were built on orthogonal partial least-square discriminant (OPLS-DA). The models evaluated were tested for over fitting with methods of permutation tests. Model validity was confirmed through 200-cycle permutation testing, with quality assessment using R^2^ and Q^2^ values. The discriminating metabolites were obtained using a statistically significant threshold of variable influence on projection (VIP) values obtained from the OPLS-DA model and two-tailed Student’s *t* test (*p* value) on the normalized raw data at univariate analysis level. The *p* value was calculated by one-way analysis of variance (*ANOVA*) for multiple groups analysis. Statistically significant metabolites were identified using dual criteria of variable importance in projection (VIP) > 1 and *p* < 0.05.

#### 4.3.5. KEGG Enrichment Analysis

To identify the perturbed biological pathways, these SAMs underwent comprehensive expression pattern evaluation and pathway enrichment analysis following established metabolomic reporting standards [53]. KEGG enrichment analyses were carried out with the hypergoemetric test, and FDR correction for multiple testing was performed. Enriched KEGG pathways were nominally statistically significant at the *p* < 0.05 level.

### 4.4. Transcriptomic Profiling and Differential Gene Expression Analysis

#### 4.4.1. Sample Preparation and RNA Sequencing

Muscle tissues from 12 fish per group (C and E) were homogenized for total RNA extraction using TRIzol Reagent (TIANGEN Biochemical Technology Co., Beijing, China) following manufacturer protocols. RNA quality and concentration were verified using a Bioanalyzer 2100 (Agilent Technologies, Santa Clara, CA, USA). Equal quantities of RNA from three biological replicates per group were pooled to generate four composite samples per group (eight total). mRNA was enriched via oligo(dT)-attached magnetic beads (Thermo Fisher Scientific, Waltham, MA, USA), fragmented, and reverse-transcribed into double-stranded cDNA using the NEBNext Ultra II RNA Library Prep Kit (New England Biolabs, Ipswich, MA, USA). Libraries were sequenced on the Illumina NovaSeq 6000 platform (150 bp paired-end reads).

#### 4.4.2. Reference Genome and Bioinformatics Pipeline

Reads were aligned to the *Oreochromis niloticus* reference genome (GCF_001858045.2; NCBI Assembly ID: 391053) using HISAT2 v2.2.1. Gene expression levels were quantified as FPKM (Fragments Per Kilobase Million) values. Differential expression analysis was performed using edgeR v3.8.2 with thresholds of |*log*2 (*fold change*)| > 1 and adjusted *p*-value (*FDR*) < 0.05.

#### 4.4.3. Functional Enrichment Analysis

GO and KEGG pathway enrichment analyses were conducted for DEGs using clusterProfiler v4.0. Statistical significance was defined as *FDR* < 0.05.

### 4.5. RT-qPCR

The gene of *GAPDH* was used as a reference gene. Gene-specific primers were created based on 11 randomly selected genes (Table 9). RT-qPCR verified the expression pattern of the 11 expressed transcripts from the Tilapia muscle tissue. FastKing gDNA Dispelling RT SuperMix and SuperReal PreMix Plus (Tiangen Biotech Co., Ltd., Beijing, China) mRNA detection assays were used for mRNA tests. Three biological replicates and triple reactions for each sample were used for all qPCR validations. The relative expression of each gene was calculated by *2^−ΔΔCt^* method, and the *log*2 value of multiple expression between the two groups was calculated.

### 4.6. Combination of Transcriptomic and Metabolomic Research

SAMs were derived from metabolomic data, whereas DEGs were obtained from transcriptomic data, according to metabolomic and transcriptomic data. Association analysis was conducted using integrated pathway analysis on genes and metabolites.

## 5. Conclusions

This study demonstrates that long-term (180-day) feeding with a faba bean-based diet (FBD) induces a comprehensive metabolic reprogramming in Nile tilapia, leading to a trade-off between significantly improved muscle textural quality and adverse effects on growth and metabolism.

On one hand, FBD feeding effectively enhanced muscle texture by promoting a hyperplasia-driven remodeling process. This was evidenced by significantly increased hardness, gumminess, and chewiness, underpinned by elevated intramuscular fat (reaching an optimal 2%), collagen content, and muscle fiber density. Metabolomic and transcriptomic analyses provided mechanistic insights, revealing that this improvement was coordinated by the upregulation of key metabolites, including ARA, and driven by the genetic regulator *chac1*, which promotes myoblast proliferation and differentiation.

On the other hand, these textural benefits were accompanied by significant trade-offs: suppressed growth performance, inhibited liver development potentially linked to taurine deficiency, reduced muscle water-holding capacity, and a broad downregulation of free amino acids and energy metabolism pathways.

In summary, our findings establish that faba bean-induced muscle quality enhancement is a multifaceted process, orchestrated through the integrated regulation of lipid metabolism (particularly ARA), collagen deposition, and the pro-hyperplastic action of the *chac1* gene. For practical application, future research should focus on dietary strategies—such as nutrient balancing or supplemental interventions—aimed at mitigating the observed growth and metabolic drawbacks while preserving and optimizing the valuable textural improvements.

## Figures and Tables

**Table 1:** Comparative effects of FBD treatments on growth performance and morphometric indices in Nile tilapia (*Oreochromis niloticus*).

Parameter (Unit)	Group C	Group E
Body weight (g)	1564.6 ± 28.2 **	1339.9 ± 49.6
Standard length (cm)	33.63 ± 0.28	32.91 ± 0.33
Total length (cm)	40.10 ± 0.30 *	39.03 ± 0.38
Condition factor (%)	4.17 ± 0.12 **	3.75 ± 0.08
Filet yield (%)	24.28 ± 0.78	25.33 ± 0.96
Visceral somatic index (%)	7.23 ± 0.22	8.23 ± 0.40
Hepatic somatic index (%)	1.60 ± 0.07 *	1.17 ± 0.13
Intestinal somatic index (%)	2.35 ± 0.16	2.60 ± 0.26
Intestinal fat rate (%)	1.99 ± 0.21	3.09 ± 0.27 *

Note: Data are presented as mean ± SE (*n* = 6). Significant differences are denoted as * *p* < 0.05 and ** *p* < 0.01 (two-tailed independent *t*-test). The same applies to the following tables.

**Table 2:** Effects of FBD treatments on physicochemical key muscle quality indices in Tilapia.

Parameter (Unit)	Group C	Group E
Muscle fiber density (n/mm^2^)	180.89 ± 8.62	231.79 ± 11.21 **
Steaming loss (%)	19.91 ± 0.95	40.12 ± 1.84 **
pH	6.55 ± 0.03 **	6.38 ± 0.00

Note: Significant differences are denoted as ** *p* < 0.01.

**Table 3:** Effects of FBD treatments on textural properties of Tilapia muscle.

Parameter (Unit)	Group C	Group E
Hardness (N)	18.89 ± 0.79	42.59 ± 3.88 **
Adhesiveness (N·mm)	0.44 ± 0.03 **	0.28 ± 0.03
Springiness (mm)	2.67 ± 0.09 **	2.33 ± 0.18
Gumminess (N)	5.29 ± 0.16	11.38 ± 1.02 **
Chewiness (mJ)	14.10 ± 0.59	26.48 ± 2.33 **

Note: Significant differences are denoted as ** *p* < 0.01.

**Table 4:** Effects of FBD treatments on proximate composition of Tilapia muscle.

Parameter (Unit)	Group C	Group E
Moisture (g/100 g)	76.88 ± 0.50	77.10 ± 0.48
Ash (g/100 g)	1.17 ± 0.02 **	1.02 ± 0.02
Protein (g/100 g)	20.03 ± 0.43	20.05 ± 0.38
Fat (g/100 g)	1.30 ± 0.15	2.02 ± 0.32 *
Energy (kJ/100 g)	408.67 ± 12.62	416.33 ± 16.04
Ca (mg/kg)	98.03 ± 1.56	91.33 ± 3.50
P (mg/kg)	2.01 ± 0.02 *	1.79 ± 0.06
Collagen (μg/mg)	0.66 ± 0.07	0.98 ± 0.09 *

Note: Significant differences are denoted as * *p* < 0.05 and ** *p* < 0.01.

**Table 5:** Comparative analysis of muscle amino acid profiles between group C and E.

Amino Acid (g/100 g)	Group C	Group E
Aspartic acid	1.88 ± 0.04 **	1.70 ± 0.03
Threonine	0.84 ± 0.02	0.78 ± 0.01
Serine	0.75 ± 0.03	0.69 ± 0.02
Glutamic acid	2.98 ± 0.08 *	2.70 ± 0.06
Proline	0.66 ± 0.01	0.61 ± 0.02
Glycine	0.95 ± 0.01	0.84 ± 0.03
Alanine	1.15 ± 0.02	1.04 ± 0.03
Cystine	0.18 ± 0.01	0.20 ± 0.00
Valine	0.91 ± 0.03	0.84 ± 0.02
Methionine	0.64 ± 0.02 **	0.55 ± 0.02
Isoleucine	0.85 ± 0.02 *	0.77 ± 0.02
Leucine	1.48 ± 0.04 *	1.34 ± 0.03
Tyrosine	0.60 ± 0.02 *	0.54 ± 0.01
Phenylalanine	0.76 ± 0.02 *	0.68 ± 0.01
Lysine	1.76 ± 0.04 *	1.60 ± 0.03
Histidine	0.46 ± 0.01 *	0.42 ± 0.01
Tryptophan	0.15 ± 0.01	0.16 ± 0.01
Arginine	1.23 ± 0.02 *	1.12 ± 0.03
Total amino acids (%)	18.33 ± 0.39 *	16.59 ± 0.40

Note: Significant differences are denoted as * *p* < 0.05 and ** *p* < 0.01.

**Table 6:** Transcriptome sequencing statistics for tilapia muscle samples.

Sample	Raw Reads	Clean Reads	Q30 (%)	Total Mapped	Mapped to Exon
C1	41,571,478	38,380,328	94.8	94.45%	98.11%
C2	42,124,554	38,892,918	94.52	93.77%	98.05%
C3	43,108,144	39,972,674	94.27	93.46%	98.12%
C4	42,147,254	38,805,278	94.71	93.74%	98.18%
E1	40,683,622	37,604,882	94.97	93.52%	97.96%
E2	42,944,138	39,714,128	94.8	94.21%	98.16%
E3	41,975,002	38,884,452	94.68	94.05%	97.96%
E4	42,402,932	39,219,948	94.71	94.53%	97.97%

**Table 7:** Comparative analysis of shared KEGG pathways between T and M profiles.

Shared Pathway	T *p*-Value	T DEGs	M *p*-Value	DAMs
Glutathione metabolism	0.015	*chac1*↑, *LOC100695721*↓	0.037	ARA↓, Ornithine↓
Sulfur metabolism	0.022	*LOC100698874*↑	0.236	Taurine↓
Taurine and hypotaurine metabolism	0.049	*cdo1*↑	0.178	Taurine↓

Note: T represent transcriptomic, while M represent tmetabolomic. ↑, up-regulated; ↓, down-regulated (DEGs/DAMs).

**Table 8:** Proximate Composition of Diet in the groups C and E.

Nutrient Component	C Group (%)	E Group (%)
Crude Protein	32.10	30.10
Crude Fat	7.90	7.34
Crude Fiber	4.20	9.80
Crude Ash	9.20	7.31
Calcium	1.58	0.51
Available Phosphorus	0.96	0.59
Lysine	1.90	1.47
Methionine	0.59	0.45
Threonine	1.20	1.06
Arginine	1.92	1.54
Tryptophan	0.28	0.39

**Table 9:** Primers used in real-time quantitative PCR.

Primer	Sequence (5′-3′)
*gapdh-F*	TGATGAGCACAGTTCACGCC
*gapdh-R*	GGGATGACTTTGCCGACAGC
*smox-F*	AGGTGGAAAGTTGCGAAAGC
*smox-R*	ATGCTGGGCTGAGTAGTTCC
*otc-F*	CCTTACAGGAGCATTACGGA
*otc-R*	CTTTGAGCCTCTTTTTCTTC
*psph-F*	ATAACAGACCATCCACCTCA
*psph-R*	CTCTCATCAAAACCAGCGTA
*ass1-F*	TCCCTGTGCCTGTGACCCCT
*ass1-R*	ACTCCGTGTTTTCCCCCGAT
*cmpk2-F*	TATCACCCATCTACTCTCAA
*cmpk2-R*	GTAGTCTTACCTGTGGCATC
*agtr1-F*	TCTACACCAGCATCTTCTTC
*agtr1-R*	CTTTCAGCCACATTTCATTT
*adcy8-F*	AGGTCACGGACGAAACACGA
*adcy8-R*	GCGGCGAAGGAAGTCATTGC
*LOC100534578-F*	TCTTTGTTTAGCAGGTGTCC
*LOC100534578-R*	TTCCTCATCTTCATTTTCGT
*LOC100702411-F*	TTTCCGTGAGCCAATCCTTT
*LOC100702411-R*	TTCCCATCCCACAACCTCCT
*cad-F*	F:CACCAGTCAGAATCACGGCT
*cad-R*	R:TTGGGGAACCAGTGAAAGTG
*egr1-F*	AGGTTCTCTCACTCCCCCAT
*egr1-R*	TCTGCTCCACCACTGGCTTC

## Data Availability

The authors confirm that the data supporting the findings of this study are available within the article. The raw sequence data of transcriptome reported in this paper have been deposited in the Genome Sequence Archive (Genomics, Proteomics & Bioinformatics 2021) in National Genomics Data Center (Nucleic Acids Res 2022), China National Center for Bioinformation/Beijing Institute of Genomics, Chinese Academy of Sciences (GSA: CRA021826) that are publicly accessible at https://ngdc.cncb.ac.cn/gsa (accessed on 3 November 2025). The raw metabolomics data have been deposited in the OMIX database at the China National Center for Bioinformation/Beijing Institute of Genomics, Chinese Academy of Sciences (accession No. OMIX008551; accessible via https://ngdc.cncb.ac.cn/omix/preview/oHZstv6s (accessed on 3 November 2025)).
